# Effects of kisspeptin incubation on the mature mouse testicular sperms quality: An experimental study

**DOI:** 10.18502/ijrm.v20i4.10903

**Published:** 2022-05-23

**Authors:** Masoumeh Akmali, Roghayeh Yalmeh, Tahereh Talaei-Khozani, Fatemeh Karimi, Elham Aliabadi

**Affiliations:** ^1^Biochemistry Department, School of Medicine, Shiraz University of Medical Sciences, Shiraz, Iran.; ^2^Anatomy Department, School of Medicine, Shiraz University of Medical Sciences, Shiraz, Iran.; ^3^Histomorphometry and Stereology Research Center, Shiraz University of Medical Sciences, Shiraz, Iran.

**Keywords:** Testis, Spermatozoa, Kisspeptin, Lactate dehydrogenase, Acrosome.

## Abstract

**Background:**

Sperm quality has an important role in the success of assisted reproductive techniques, by adding some bioactive agents with a positive impact on sperms, it can be improved.

**Objective:**

This study aimed to evaluate the effects of kisspeptin on the sperm motility criteria, Lactate dehydrogenase-C (LDHC) activity, acrosomal reaction, and capacitation in the mouse testicular sperm in vitro.

**Materials and Methods:**

Sperm samples were extracted from testes of 96 male Balb/C mice weighing 25-30 gr, aged 6-8 wk. Then, they were separated into 4 parts; 2 controls and 2 kisspeptin-treated aliquots; each one incubated for either 15 or 30 min. The sperm motility and the LDHC activity were evaluated, and also the frequency of the non-capacitated, intact, and acrosomal-reacted sperms were evaluated by staining with Wheat germ agglutinin, Peanut agglutinin, and Concanavalin A, respectively. The stained sperms were analyzed by flow cytometry and fluorescent microscope.

**Results:**

Our result showed that kisspeptin increased both the sperm motility (p = 0.04) and LDHC enzyme activity (p = 0.04) after 15 min of incubation. At the same time, it did not impact the frequency of the non-capacitated, intact and acrosomal-reacted sperms after incubation in the same period (p = 0.16).

**Conclusion:**

A 15 min period of incubation with kisspeptin could be applicable for evaluating sperm motility and LDH activity.

## 1. Introduction

A correlation has been shown between fertility outcomes and sperm quality. Acrosomal function (1), sperm motility and morphology (2), and sperm DNA fragmentation index (3) are some of the sperm features which are correlated with fertility rate and clinical pregnancy. Therefore, it can be helpful to use a high-quality sperm population in each assisted reproductive technique (ART) to improve its outcomes. Some studies have focused on the improvement of sperm quality by adding supplements to ejaculated sperms and testicular extraction before ART applications (2, 4-7).

In some cases, such as non-obstructive azoospermia, the sperms are too low to consider the patient a fertile man. Azoospermia is the cause of infertility in 20% of men (8), and 60% of them have non-obstructive azoospermia (9). Spermatogenesis in non-obstructive azoospermia men is limited; however, testicular sperm extraction is an appropriate method for men who suffer from non-obstructive azoospermia and the other types of azoospermia (10). Extracted sperms from testis are immature and contain abnormal sperms (11). Therefore, it is possible to improve testicular sperm quality by adding bioactive additives.

Kisspeptin is a regulator of the reproductive system with a wide range of functions (12). Kisspeptin injection has been reported to improve semen quality, including sperm concentration and viability, and the production of testosterone in rheotaxis (13). Kisspeptin has been detected in epididymal epithelium, fallopian tube ciliated epithelial cells, and cumulus-oocyte complex, while its receptor expressed in the acrosomal region of both spermatids and mature spermatozoa (14), and it is well known that mammalian sperms earn fertilization capability during transfer through epididymis (15) and the female reproductive system (16). Kisspeptin has been detected in mice Leydig and Sertoli cells (17). Spermatids and mature spermatozoa have a receptor for kisspeptin and are also exposed to the various cells which synthesize kisspeptin during their journey from somniferous tubules to female reproductive tracts. Therefore, kisspeptin can be considered a part of the spermatid and sperm microenvironment, and it can be hypothesized that kisspeptin is involved in their maturation. Besides, adding a kisspeptin antagonist to the sperm extracted from epididymis can decrease in vitro fertilization rate (14). Therefore, it was assumed that kisspeptin supplementation to the sperm environment might help improve sperm quality.

Reproduction regulation in adult animals is a complex process that needs the cooperation of the forebrain, pituitary gland, and gonads (18). Kisspeptin and its receptor, Kisspeptin receptor (GPR54), upstream of the gonadotropin-releasing hormone neuronal network, have a role in controlling the reproductive axis (19). Disturbance in kisspeptin signaling leads to hypogonadotropic hypogonadism in mice and humans (20). Kisspeptin and GPR54 are expressed in various tissues such as the placenta, kidney, heart, pancreas, and uterus (2, 21-24). Besides kisspeptin, GPR54 has been found in humans and mice testis (19).

The current study was designed to investigate the effects of kisspeptin as part of the natural sperm microenvironment that is isolated from testicular sperm extraction methods for treating azoospermia cases recapitulate the natural sperm condition in reproductive tracts.

## 2. Materials and Methods

### Animals

In this experimental study, 8-wk-old mice weighing 37 gr were placed in standard cage with 12 hr light/dark cycle, and free access to food and water ad libitum.

### Sperm preparation 

The mouse testes were removed under deep anesthesia, and minced using 2 insulin needles to free the sperms from seminiferous tubules. The extracted sperms remained at room temperature for 1 hr with occasional shaking. Finally, the sperms were centrifuged at 1000 rpm for 5 min. Leydig and Sertoli cells settled down, and sperm swam in the supernatant (25).

#### Experimental design

Sperm samples extracted from 96 mice were divided into 2 parts; the first aliquot was used for lectin histochemistry assay and the second one for enzyme assay. Each part was aliquoted into 2 subgroups. The control group was incubated with Ham's F10, and the other group was exposed to Ham's F10 (Sigma, USA) containing 75 µM of Kisspeptin (Sigma, USA) for 15 min. Then, sperm motility was checked.

### Sperm motility

Sperm motility was checked at the beginning and the end of the experiments according to world health organization criteria. Types of sperm motility, including immotile, non-progressive and progressive, were checked (26).

#### Enzyme assessment

The second part of the samples was sonicated and centrifuged at 13500 rpm for 20 min. To assess the lactate dehydrogenase C enzyme activity, phosphate buffer, pyruvate (Sigma, USA), and Nicotinamide adenine dinucleotide + hydrogen (Sigma, USA) were added to the test samples. The blank solution is composed of phosphate buffer saline, pyruvate, and sperm lysate. The optical density of the blank and the tests were then checked with spectrophotometry at 345 nm wavelength.

#### Lectin histochemistry

We used wheat germ agglutinin (WGA), peanut agglutinin (PNA), and concanavalin A (Con A) lectins (all from Sigma, USA) to check the frequency of the capacitated, acrosomal intact, and acrosomal reacted sperms. The samples were centrifuged at 1000 rpm for 5 min. Then, the supernatant was discarded, and the pellet was resuspended in 2% paraformaldehyde (Merck, Germany) for 30 min. After washing the samples with phosphate buffer saline, they were incubated in appropriate lectins at dilution of 10 µg/mL for 2 hr in the dark. The percentage of reacted sperms and the intensity of the reactions were analyzed by flow cytometry. The data was also analyzed by flowJo-X software (25). A sample of the sperms was also prepared for morphological examination by fluorescent microscopy. The procedure was the same as that performed for flow cytometry; however, the samples were counterstained with Hoechst to detect the nuclei at the end of the procedure.

### Ethical considerations

Experimental procedures were performed under the authority of a Home Office Project License and approved by the Ethics Committee of the Shiraz University of Medical Sciences, Shiraz, Iran (Code: IR.SUMS.REC.1396.S181).

### Statistical analysis

All results were shown as mean ± SE. All of them were analyzed by One-way ANOVA and the Least Significant Difference test using Statistical Package for the Social Sciences software (SPSS) version 23 for windows (IBM, USA). In addition, diagrams were drawn by Graph Pad Prism (version 6.07). Statistical significance was defined at p < 0.05.

## 3. Results

### Sperm motility assay

**Figure 1:**
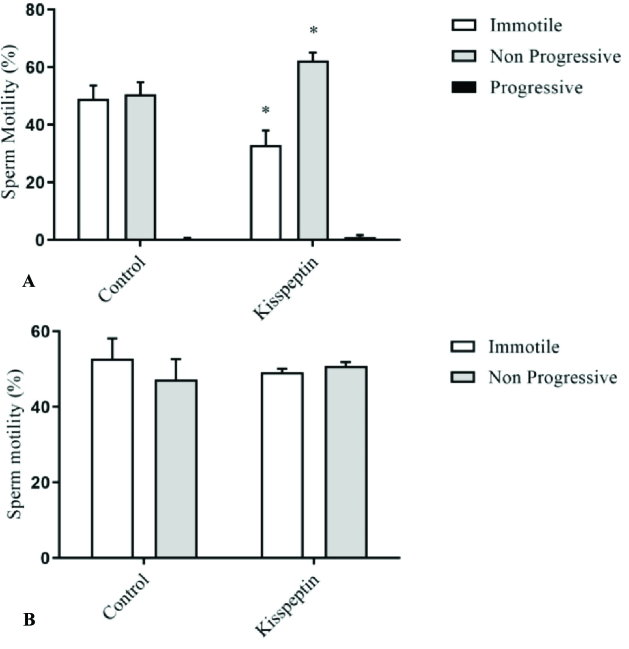
The graph shows the comparison of the sperm motility in the samples incubated in kisspeptin and conventional media (control) after 15 (A) and 30 (B) min. Kisspeptin led to a significant increase in the frequency of the non-progressive motile sperms and decrease immotile sperms after 15 min. *Significant different with control (p < 0.05).

Evaluation of the motility showed a significant increase in the percentage of the non-progressive sperms and a significant decrease in the percentage of the immotile sperms in kisspeptin-treated samples compared to the control at 15 min after exposure. After 30 min of incubation, kisspeptin could not change the sperm motility significantly (Figure 1).

### Lactate dehydrogenase-C (LDHC) activity 

**Figure 2:**
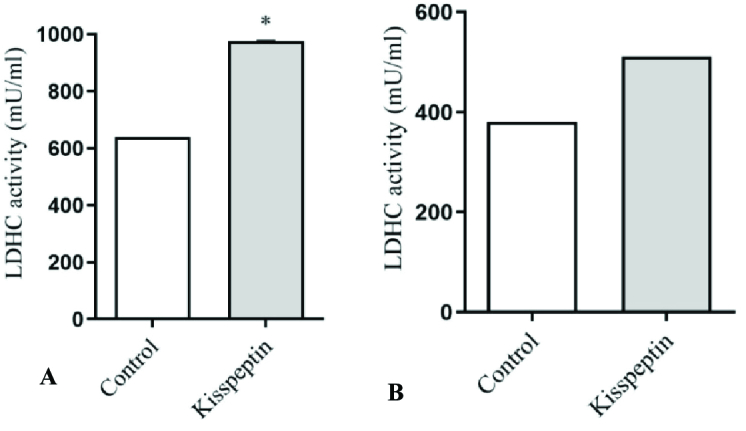
The comparison of the LDHC activity in the samples incubated in kiss peptin and conventional media (control) after 15 (A) and 30 (B) min. LDHC activity increased significantly after 15 min incubation with kisspeptin. *Significant different with control (p < 0.05) LDHC: Lactate dehydrogenase-C.

LDHC activity, as an indicator of sperm motility, showed a significant elevation in kisspeptin-treated samples after 15 min. In line with motility, no significant difference was detected in LDHC activity after 30 min of incubation with kisspeptin (Figure 2).

### Lectin histochemistry

All lectins, including PNA, WGA, and Con A, reacted with the acrosomal region of testicular sperms. Data demonstrated the distribution pattern of glycoconjugates in kisspeptin-treated samples was similar to those in control samples.

### Capacitation

**Figure 3:**
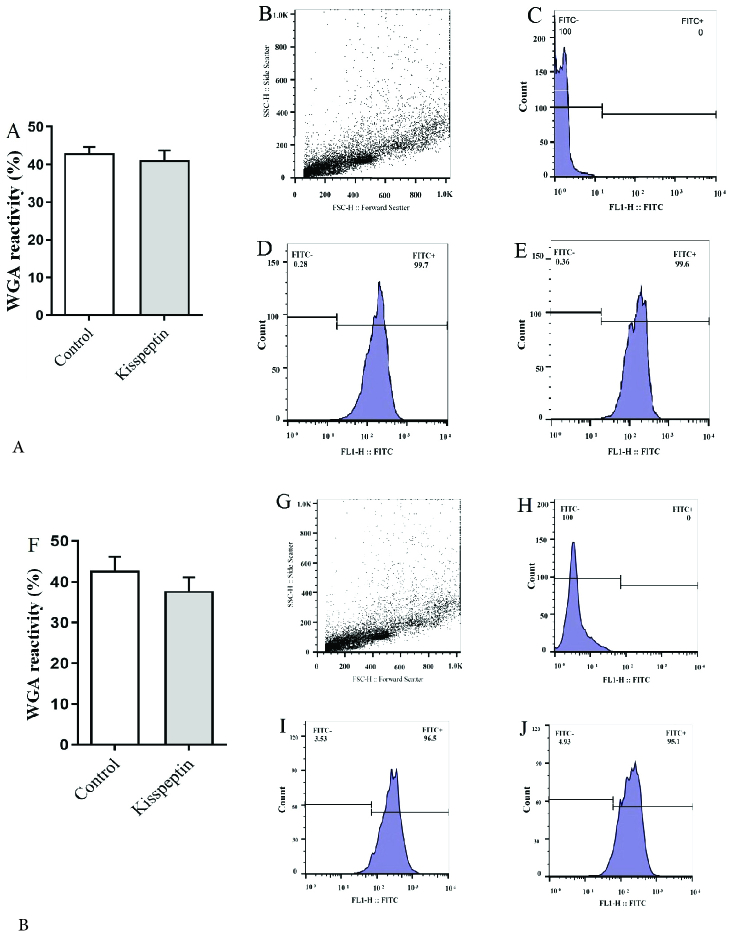
Comparison of the frequency of the capacitated sperms stained with WGA. Graph (A) shows that the frequency of the capacitated sperms was similar in both control and kisspeptin-treated samples after 15 min. A represented flow cytometry graph sample, B: Dot plot, C: Unstained, D: Control, and E: Kisspeptin-treated samples. F-J shows counterpart samples after 30 min. *Significant difference with control (p < 0.05) WGA: wheat germ agglutinin, FITC: Fluorescein isothiocyanate.

In both incubation periods, 15 and 30 min exposure to kisspeptin did not significantly influence the percentage of WGA-reacted sperms. Flow cytometry revealed the mean of fluorescent intensity also follows the same trend (Figure 3).

### Acrosomal reaction

**Figure 4:**
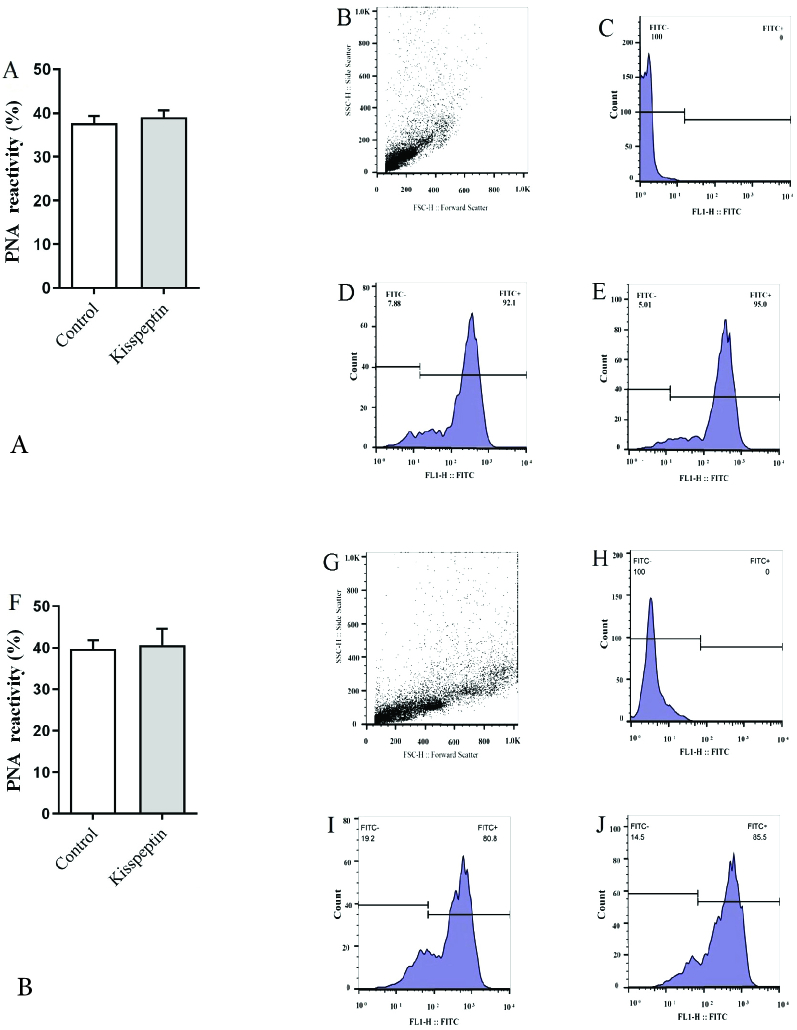
Effect of kisspeptin on the testicular acrosome-intact sperms that stained with PNA. The graph (A) shows the percentage of the sperms with intact acrosome was similar in both control and kisspeptin-treated samples after 15 min. A: Sample of represented flow cytometry graph, B: Dot plot, C: Unstained, D: Control and E: Kisspeptin-treated samples. F-J shows counterpart samples after 30 min. Significant different with control (p < 0.05), PNA: peanut agglutinin; FITC: Fluorescein isothiocyanate.

**Figure 5:**
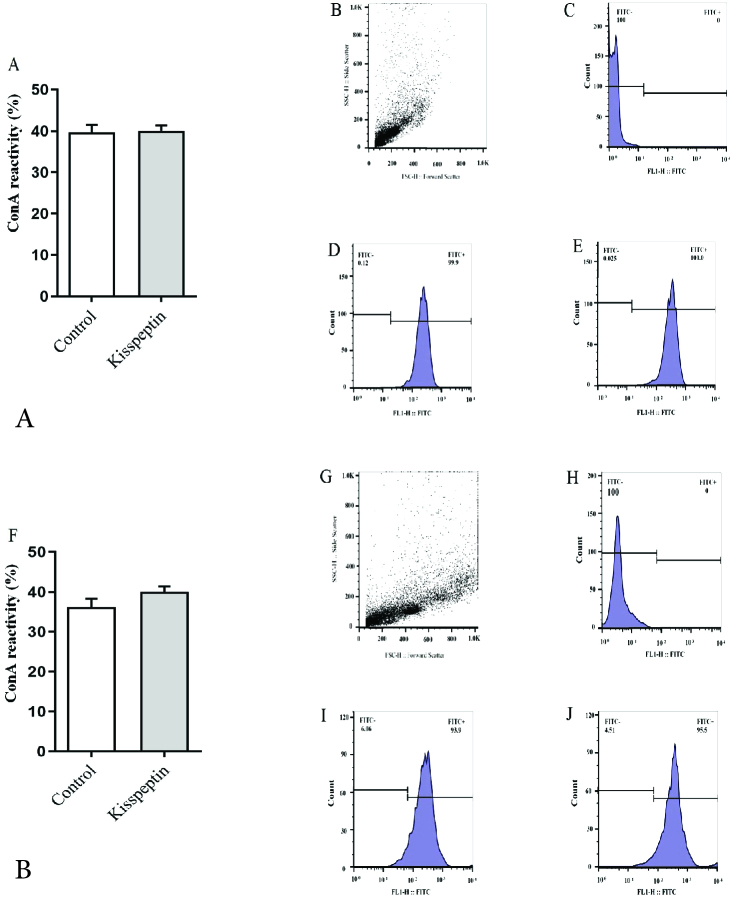
Effects of kisspeptin on the acrosome reaction that detected by Con-A. Flow cytometry analysis of samples shows that kisspeptin did not impact in the percentage of sperms with spontaneous acrosome reaction. Also, the time of incubation did not influence on the frequency of acrosomal-reacted sperms. The graph (A) compares the frequency of acrosome-reacted samples in the both control and kisspeptin-treated samples after 15 min. A sample of represented flow cytometry graph, B: Dot plot, C: Unstained; D: Control and E: Kisspeptin-treated samples. F-J shows counterpart samples after 30 min. Significant different with control (p < 0.05), FITC: Fluorescein isothiocyanate.

**Figure 6:**
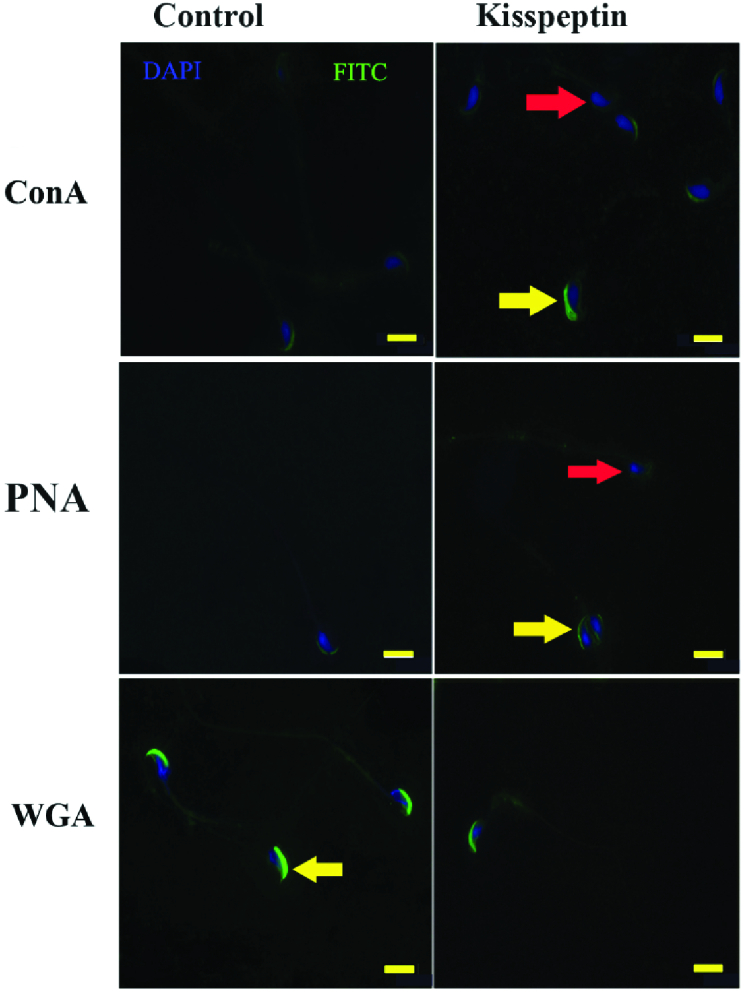
Lectin histochemistry revealed the distribution patterns of the reaction with Con A, PNA and WGA in head of the sperms. Con A reacted with the mannose residues at posterior acrosomal membrane in the acrosomal reacted-sperms (yellow arrow). Red arrow shows the acrosomal intact sperms. PNA stained with acrosomal content and the acrosomal intact sperms react with PNA at the anterior pole of the nucleous where the acrosome located (yellow arrow). The sperms without PNA reactivity are spontaneously acrosomal reacted (red arrow). The non-capacitate sperms also react with WGA (yellow arrow in the bottom pictures). Scale bar is 10 µm, PNA: peanut agglutinin; WGA: wheat germ agglutinin.

PNA was used to detect the intact acrosome. Kisspeptin did not affect the frequency of the sperms with intact acrosome regardless of incubation time. The mean of fluorescent intensity was also similar in kisspeptin- and control-exposed samples (Figure 4). Con A staining showed that the acrosomal reaction occurred in sperms and the posterior membrane of the acrosome, where it contains a lot of Con A-reacted residues, exposed to the surface. Flow cytometry revealed the frequency of the acrosomal-reacted sperms was statistically similar in the kisspeptin-treated and control samples, and the incubation time had no effect on the result. The mean fluorescent intensity confirmed that the kisspeptin had no significant effect on the acrosomal reaction (Figure 5). The distribution pattern of the different lectins reacted residues was also demonstrated in (Figure 6). The acrosomal region reacted with WGA in the head region of the non-capacitated sperms. Also, the intact acrosome reacted with PNA. The posterior membrane of the acrosome of the sperms stained with Con A was demonstrated in the acrosome reaction (Figure 6).

## 4. Discussion

To our knowledge, the effects of kisspeptin on testicular sperms were not reported. Sperms acquire the ability to fertilize the oocytes while passing through the epididymis and female reproductive system. This ability is considered a critical factor for the successful rate of ART procedures (14, 27), especially in techniques that use sperms extracted from testis (25). The beneficial influence of kisspeptin on sperm hyperactivation has been reported, which is characterized by an increase in the amplitude of lateral head displacement and beat cross frequency, and a decrease in the straightness and linearity movement (16). The data from the current study also showed that kisspeptin administration for 15 min improved motility and LDHC activity. At the same time, incubation of the samples for a more extended period had no impact on these 2 criteria. A possible mechanism may be attributed to the elevation of intracellular calcium by kisspeptin incubation a few minutes after exposure with a plateau at 3-6 min (16). Intracellular calcium elevation activates a signaling pathway that leads to an increase in flagellar movement (28).

Along with this finding, we also showed sperm motility elevation after 15 min incubation with kisspeptin. On the other hand, long exposure time (30 min) of kisspeptin to ejaculated human samples has been reported to decrease sperm motility (16). In line with this finding, we also observed a similar trend for sperm motility in testicular sperm.

A suggested mechanism for the time-dependent effects of kisspeptin in sperm motility is the binding of kisspeptin to its receptor, which leads to activation of phospholipase C and, as a result, phosphatidylinositol 4, 5-bisphosphate cleaves to inositol 1, 4, 5 triphosphates and diacylglycerol. In turn, inositol 1, 4, 5 triphosphates bind to its receptor on the surface of mitochondria and acrosome (29). Finally, the first phase of calcium elevation happens. For the second phase of calcium elevation, sperms need exogenous calcium and kisspeptin continuously added to the media (30). The principal part of the sperm tail calcium channels called CatSper, influx calcium into the sperm, leading to hyperactivation (31). In another study, it has been shown that hyperactivation fails to happen in CatSper knockout transgenic mice (29).

With regard to the above evidence and our data, a potential relationship between CatSper and LDHC activity may be hypothesized in the presence of kisspeptin; however, it needs more investigation in the future. Kisspeptin can also be considered an antioxidant (32). This might be the other mechanism that may justify the beneficial impact of kisspeptin on sperm quality. Reactive oxygen species (ROS) are produced during different ART procedures (33), including sperm extraction from the testis, which contains more immature sperms. Immature sperms produce more ROS than mature ones. Sperm motility has decreased under highly ROS conditions (34). Therefore, it may be hypothesized that kisspeptin acts as an antioxidant to prevent the detrimental effects of ART techniques on sperm motility.

Con A and PNA reactions indicate intact-acrosome and acrosome-reacted sperms, respectively (7). Also, WGA is a marker to detect non-capacitated and non-acrosome reacted samples (27). Our finding revealed that kisspeptin did not affect on the frequency of capacitating and acrosomal reacted sperms after 15 and 30 min. There are contradictory reports on the role of kisspeptin in capacitation and acrosomal reaction. While a report indicates the role of kisspeptin in capacitation and acrosomal reaction in ejaculated sperms (35), another study suggested that kisspeptin had no impact on acrosomal reaction (14). Our data indicates kisspeptin incubation has no significant effect on these parameters. Another reason for the contradictory results may be the sperm source and the state of maturity. As sperm cross through the epididymis, the GPR54 translocate from the acrosomal membrane to the plasma membrane. In addition, kisspeptin is detected in the epididymal epithelium (14). Therefore, kisspeptin can impress differentially in sperms from various sources. This may be due to the shortage of GPR54s at the plasma membrane of testicular sperm, it could not influence the capacitation and acrosomal reaction. Besides, it seems that the duration of kisspeptin exposure is the critical factor for capacitation and then acrosomal reaction.

Different doses and durations of kisspeptin incubation are investigated in the human ejaculated sperms. Also, isolated capacitate sperms were treated with kisspeptin for 30, 60, and 120 min. It has been shown that the percentage of the acrosomal reacted sperms decreased in the presence of kisspeptin with respect to kisspeptin antagonist-treated samples (16). In the present study, the incubation time was different from the aforementioned study. As the duration of the incubation with kisspeptin has a critical role in its activities, the contradictory result may be attributed to the differences in incubation time. Also, the aforementioned study was conducted on the epididymal capacitated sperms rather than whole testicular sperms.

## 5. Conclusion 

The current study data indicated that after 15 min, both motility and LDH activity elevated significantly by incubating the sperms in a medium containing kisspeptin. At the same time, it did not influence sperm capacitation and acrosomal reaction.

##  Conflict of Interest

No conflict of interest.
